# Evaluating Photosynthetic Light Response Models for Leaf Photosynthetic Traits in Paddy Rice (*Oryza sativa* L.) Under Field Conditions

**DOI:** 10.3390/plants14010023

**Published:** 2024-12-25

**Authors:** Xinfeng Yao, Huifeng Sun, Sheng Zhou, Linyi Li

**Affiliations:** 1 Institute of Agricultural Science and Technology Information, Shanghai Academy of Agricultural Sciences, Shanghai 201403, China; xinfengyao@saas.sh.cn; 2 Key Laboratory of Intelligent Agricultural Technology (Yangtze River Delta), Ministry of Agriculture and Rural Affairs, Shanghai 201403, China; 3 Eco-Environmental Protection Research Institute, Shanghai Academy of Agricultural Sciences, Shanghai 201403, China; sunhuifeng@saas.sh.cn (H.S.); zhous@outlook.com (S.Z.); 4 Shanghai Engineering Research Centre of Low-Carbon Agriculture (SERCLA), Shanghai 201415, China; 5 Key Laboratory of Low-Carbon Green Agriculture in Southeastern China, Ministry of Agriculture and Rural Affairs, Shanghai 201403, China

**Keywords:** light-response curves, light acclimation, photosynthetic parameters, paddy rice (*Oryza sativa* L.), Ye model

## Abstract

Accurate photosynthetic parameters obtained from photosynthetic light-response curves (LRCs) are crucial for enhancing our comprehension of plant photosynthesis. However, the task of fitting LRCs is still demanding due to diverse variations in LRCs under different environmental conditions, as previous models were evaluated based on a limited number of leaf traits and a small number of LRCs. This study aimed to compare the performance of nine LRC models in fitting a set of 108 LRCs measured from paddy rice (*Oryza sativa* L.) grown in field across 3 years under different leaf positions, leaf ages, nitrogen levels, irrigation levels, and varieties. The shape of 108 LRCs varies significantly under a range of leaf traits, which can be typed into three leaf light-acclimation types—high-light leaves (HL-1 and HL-2), and low-light leaves (LL). The accuracy of these models was evaluated by (1) LRCs from three acclimation types: HL-1 and HL-2, and LL; and (2) LRCs across three irradiance stages: light-limited, light-saturated, and photoinhibition. Results indicate that the Ye model emerged as the top performance among the nine models, particularly in the photoinhibition stage of LL leaves, with median values of *R^2^*, *SSE*, and *AIC* of 0.99, 2.39, and −14.03, respectively. Furthermore, the Ye model produced the most accurate predictions of key photosynthetic parameters, including dark respiration (*R_D_*), light-compensation point (*I_comp_*), maximum net photosynthetic rate (*P_Nma_*_x_), and light-saturation point (*I_sat_*). Results also suggest that *P_NImax_* and *I_max_* were the most appropriate parameters to describe photosynthetic activity at the light-saturation point. These findings have significant implications for improving the accuracy of fitting LRCs, and thus robust predictions of photosynthetic parameters in rice under different environmental conditions.

## 1. Introduction

Photosynthesis is the most important biochemical process that converts sunlight into chemical energy, providing the basis for plant growth and yield [1]. However, the stagnation of rice yields in recent decades is partly associated with the low efficiency of converting light energy into biomass, highlighting the need for targeted improvements in this critical process [2,3,4]. As the world’s most important staple food crop, improving photosynthetic efficiency in rice can significantly boost productivity, directly impacting food security for millions of people worldwide [5].

To optimize photosynthesis, plants undergo photosynthetic acclimation, which involves changes in the composition, function, and structure of the photosynthetic apparatus in response to environmental conditions, particularly to incoming solar irradiance, as well as temperature, and water availability, nutrients, and CO_2_ [6,7,8,9,10]. Accurately and rapidly characterizing a light-response curve (LRC) of photosynthesis at the leaf level can provide valuable insights into this acclimation process across a broad range of light intensities [11,12]. Additionally, several key photosynthetic parameters estimated from LRCs, such as dark respiration (*R_D_*), light-compensation point (*I_comp_*), quantum yield (*ϕ*), maximum net photosynthetic rate (*P_Nmax_*), and light-saturation point (*I_sat_*), offer valuable information for assessing the photosynthetic efficiency and capacity [13,14].

Several mathematical models are utilized to describe LRCs, with the most widely used being rectangular hyperbola (RH) models [15,16,17], hyperbolic tangent (Tanh) models [18,19], non-rectangular hyperbola (NRH) models [11,20,21], exponential (Exp) models [22,23], and the Ye model [12,13].

Furthermore, although the models described above have been widely used to obtain key photosynthetic parameters by fitting LRCs [24,25,26], fewer studies have assessed the suitability, effectiveness, and accuracy of these LRC models [27]. Comparisons of LRC models have been conducted for cereal crops, including paddy rice [12,13], winter wheat [28], spring wheat [29], and summer maize [30]. Among the RH, Tanh, NRH, Exp, and Ye models, research indicates that the Ye model performs best, outperforming even the widely used NRH model [12,28,30]. However, Song et al. (2021) used LRC models to simulate the LRC of spring wheat under varying soil water contents caused by mulching treatments. In their study, the Ye model exhibited the lowest simulation accuracy, whereas the NRH model demonstrated the smallest error and provided the best simulation results [29].

Discrepancies in the model comparison results may be attributed to the limited number of LRC observations, often fewer than 10 curves, used in these studies [12,28,29,30]. Additionally, these limited observations were typically taken at a specific growth stage, usually the filling stage, and from fully expanded leaves, such as flag leaves, which were unshaded and acclimated to high light.

Previous studies have highlighted light as a dominant factor influencing the photosynthetic characteristics of leaves in mature canopies [6,8]. Irradiance levels can vary dramatically within the canopy, decreasing significantly from the top (high light, over 2000 μmol m^−2^s^−1^) to the bottom (low light, less than 100 μmol m^−2^s^−1^), which profoundly impacts photosynthesis saturation and the extent of photo-inhibition [6]. Additionally, photosynthetic characteristics are highly variable and can be influenced by leaf traits such as nitrogen content, age, and mass [6,21]. Essentially, a robust LRC model should accurately capture leaf-scale photosynthetic responses across all irradiance levels under variable leaf traits [12]. Therefore, identifying the “best” model among competing options remains challenging [14,31].

In this study, 3-year continuous field experiments were conducted to obtain a comprehensive set of LRCs in rice leaves, capturing the diversity caused by variations in leaf positions, leaf ages, nitrogen levels, irrigation levels, and varieties. This set was then used to evaluate the performance of nine LRC models under various conditions: (1) LRCs from three acclimation types: high light (HL-1 and HL-2) and low light (LL), and (2) LRCs across three irradiance stages: light-limited, light-saturated, and photoinhibition. The optimal model that best describes the LRCs and related photosynthetic parameters for rice grown in field was identified based on its goodness of fit. The findings of this study provide practical guidance on the selection of the most appropriate LRC model for characterizing diverse LRCs.

## 2. Results

### 2.1. Classification of LRCs Based on the Fitted Results

**Figure 1:**
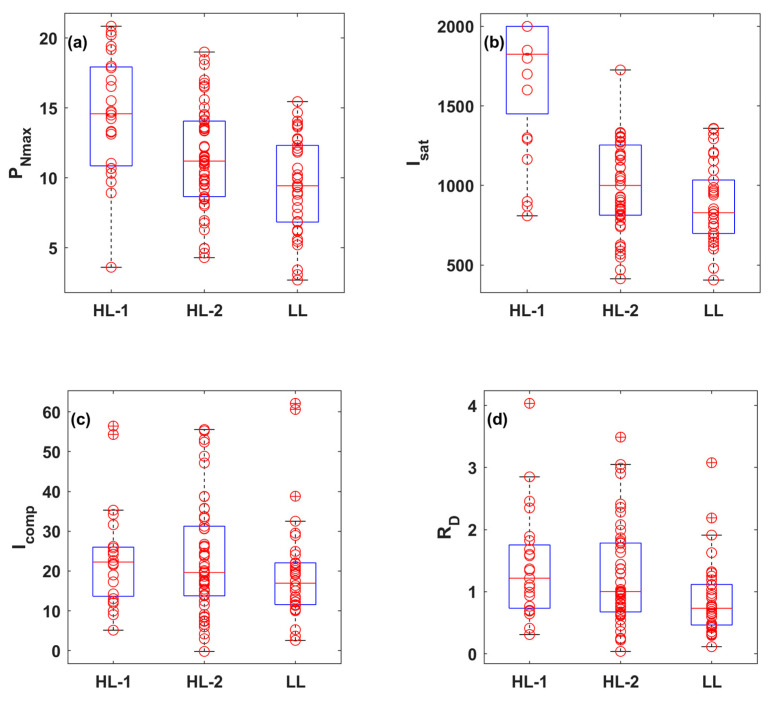
Box plots of observed values for *P_Nmax_* (**a**), *I_sat_* (**b**), *I_comp_* (**c**), and *R_D_* (**d**) derived from the LRCs in the HL-1, HL-2, and LL types. The red circles indicate the observed values, while the blue box plot shows the middle 50% of the data (interquartile range, IQR), the lower and upper boundaries correspond to the first (Q1) and third quartiles (Q3), respectively, and the red line denotes the median. The lower and upper whiskers represent the minimum and maximum values within 1.5 times the IQR from Q1 and Q3, and any points beyond the whiskers are considered outliers.

**Figure 2:**
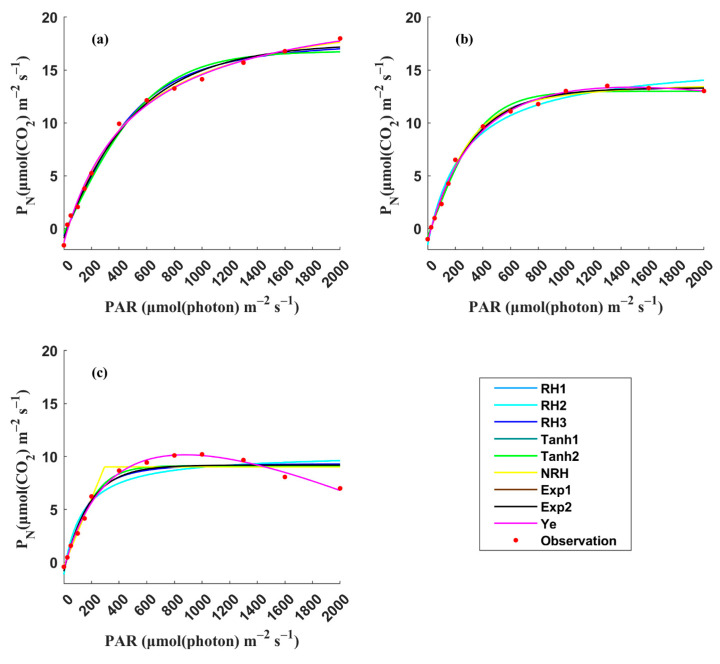
Comparisons of three representative LRCs from HL-1 (**a**), HL-2 (**b**), and LL (**c**) leaves fitted by the nine models.

The 108 LRCs were fitted using the nine LRC models mentioned above. The results showed significant variations among the LRCs under different conditions (leaf positions, leaf ages, nitrogen levels, irrigation levels, and rice varieties), which can be broadly divided into three leaf light-acclimation types: LRCs from HL-1, HL-2, and LL leaves. The observations of the four key photosynthetic parameters (*P_Nmax_*, *I_sat_*, *I_comp_*, and *R_D_*) were statistically analyzed for each leaf light-acclimation type (HL-1, HL-2, and LL) and displayed using box plots (Figure 1). Figure 2 presents three representative LRCs from HL-1, HL-2, and LL leaves, fitted by the nine models.

(1) HL-1: LRCs measured with leaves acclimated to high light, and *P_N_* showed a continuous increase with the increase of light, with median observed values of *P_Nmax_*, *I_sa_*_t_, *I_comp_*, and *R_D_* of 14.58 μmol (CO_2_) m^−2^ s^−1^, 1825 μmol (photon) m^−2^ s^−1^, 22.26 μmol (photon) m^−2^ s^−1^ and 1.22 μmol (CO_2_) m^−2^ s^−1^, respectively. All nine models were successful in accurately fitting the representative LRC for the HL-1 type.
(2) HL-2: LRCs measured with leaves acclimated to high light, and *P_N_* increased with the increase of light, eventually became light-saturated and then remained almost unchanged with additional light. The median observed values of *P_Nmax_*, *I_sat_*, *I_comp_*, and *R_D_* were 11.19 μmol (CO_2_) m^−2^ s^−1^, 1000 μmol (photon) m^−2^ s^−1^, 19.63 μmol (photon) m^−2^ s^−1^ and 1.00 μmol (CO_2_) m^−2^ s^−1^, respectively. Similarly, all nine models successfully achieved accurate fits for the representative LRC for the HL-2 type.
(3) LL: leaves acclimated to low light, and *P_N_* reached the light-saturation point at a relatively low light level and then decreased continuously with the increase of light, exhibiting significant photoinhibition. The median observed values of *P_Nmax_*, *I_sat_*, *I_comp_*, and *R_D_* were 9.43 μmol (CO_2_) m^−2^ s^−1^, 829 μmol (photon) m^−2^ s^−1^, 16.94 μmol (photon) m^−2^ s^−1^ and 0.73 μmol (CO_2_) m^−2^ s^−1^, respectively. The Ye mode accurately described the photoinhibition stage in the representative LRC for the LL type.

Furthermore, the proportions of the three types of LRCs from leaves across various nitrogen levels and leaf positions were analyzed. The three types of leaves were observed across all nitrogen levels (Figure S1a). HL-1 had the highest proportion under the N200 level (28.9%) and was relatively evenly distributed across the other three nitrogen levels. HL-2 exhibited the highest proportion, primarily found under N100 and N200 levels (over 50%). In contrast, LL was predominantly observed under N0 and N300 levels (over 40%), while it showed the lowest proportion under the N200 level (18.4%). A detailed statistical analysis of the three leaf types in relation to the leaf positions is provided in the Supplementary Material (Text S1 and Figure S1b–d).

### 2.2. Comparisons of Model Accuracy

#### 2.2.1. Performances of Overall Fitting Accuracy

**Figure 3:**
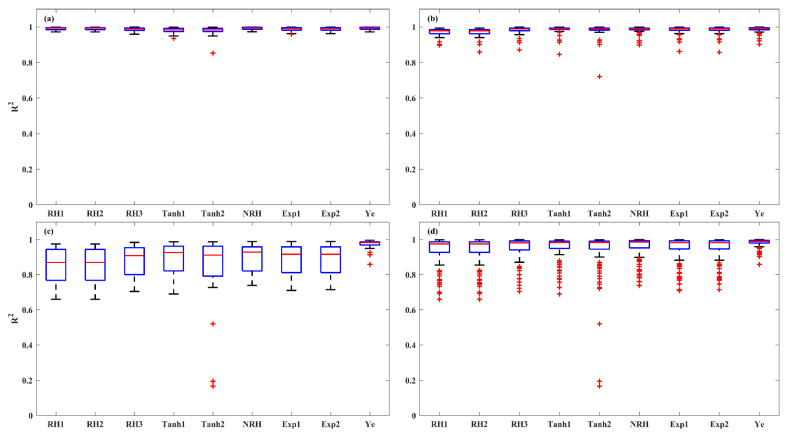
The *R^2^* of the nine models for fitting LRCs involved in the HL-1 (**a**), HL-2 (**b**), and LL (**c**) and overall set (**d**). Red + indicate outliers, defined as values beyond the minimum and maximum values within 1.5 times the IQR from the first and third quartiles.

**Figure 4:**
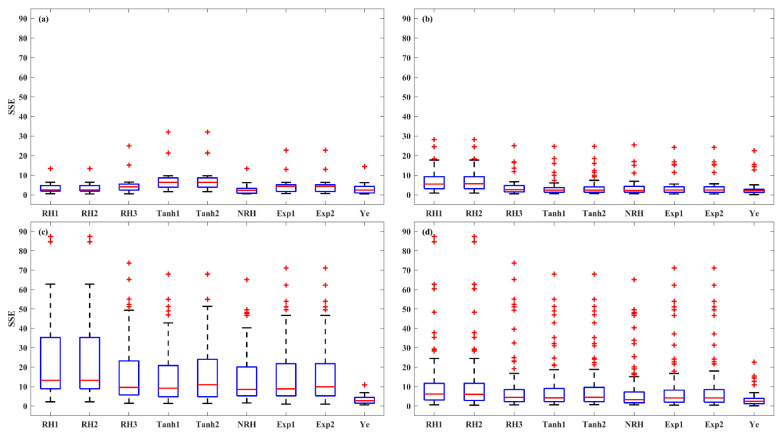
The *SSE* of the nine models for fitting LRCs involved in the HL-1 (**a**), HL-2 (**b**), and LL (**c**) and overall set (**d**). Red + indicate outliers, defined as values beyond the minimum and maximum values within 1.5 times the IQR from the first and third quartiles.

**Figure 5:**
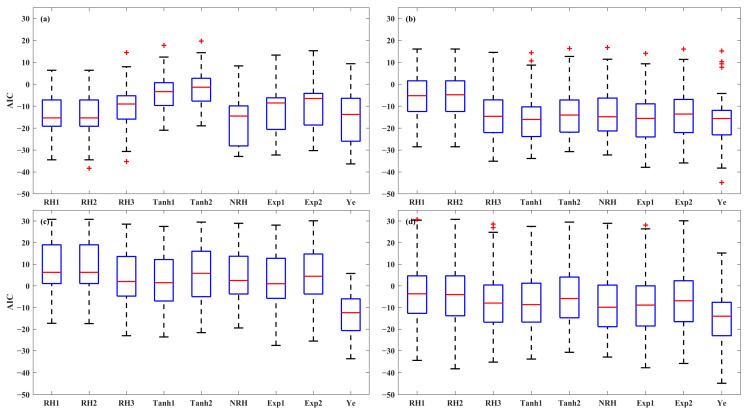
The *AIC* of the nine models for fitting LRCs involved in the HL-1 (**a**), HL-2 (**b**), and LL (**c**) and overall set (**d**). Red + indicate outliers, defined as values beyond the minimum and maximum values within 1.5 times the IQR from the first and third quartiles.

An evaluation of model accuracy among the nine LRC models was conducted for each light-acclimation type (HL-1, HL-2, and LL) and overall set. The performance of the models was assessed using the *R^2^*, *SSE*, and *AIC*, as shown in the boxplots (Figure 3, Figure 4 and Figure 5). The results indicate that the models were most successful in fitting the LRCs from HL-1 leaves, followed by those from HL-2 leaves, with the poorest fit for LL leaves. For HL-1 leaves, the RH1, RH2, NRH, and Ye models demonstrated the best performance, exhibiting high accuracy with median *R^2^* values greater than 0.99, median *SSE* below 2.52, and median *AIC* values lower than −13.76. For HL-2 leaves, all models except RH1 and RH2 demonstrated strong performance, with median *R^2^* values above 0.99, median *SSE* below 2.66, and median *AIC* values less than −13.58. For LL leaves, the performance of the first eight models in describing the photoinhibition stage of *P_N_* was limited, as evidenced by their low *R^2^* values, high *SSE*, and *AIC* values. In contrast, the Ye model delivered significantly better results in fitting the LRCs from LL leaves, with a median *R^2^* of 0.98, a median *SSE* of 2.71, and a median *AIC* of −12.39. The models for the overall set (across all three leaf types) were ranked by accuracy, with the Ye model performing the best, followed by NRH, Exp2, Exp1, Tanh1, Tanh2, RH3, RH2, and RH1. The Ye model was observed to provide the best fit for the overall LRCs, with a median *R^2^* of 0.99, a median *SSE* of 2.39, and a median *AIC* of −14.03.

#### 2.2.2. Performances of Models over Light-Limited, Light-Saturated, and Photoinhibition Stages

**Figure 6:**
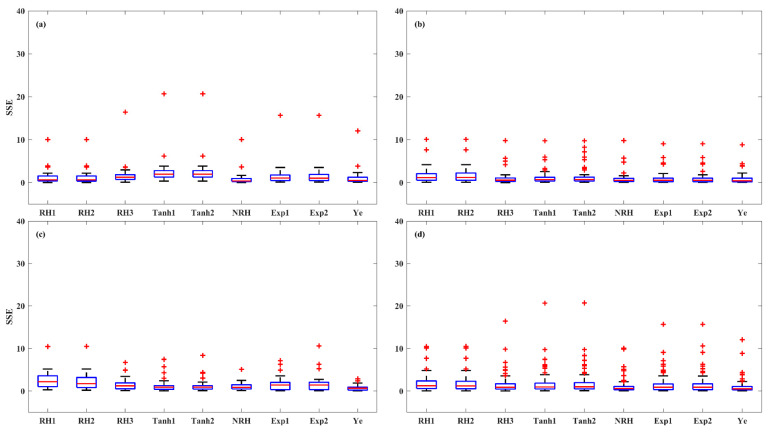
*SSE* values of the LRCs in the light-limited stage (0 and 200 μmol (photon) m^–2^ s^–1^) across HL-1 (**a**), HL-2 (**b**), and LL (**c**) and overall set (**d**). Red + indicate outliers, defined as values beyond the minimum and maximum values within 1.5 times the IQR from the first and third quartiles.

**Figure 7:**
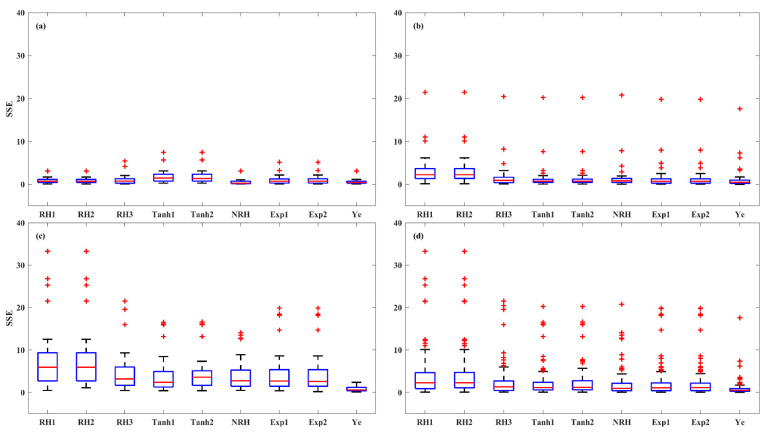
*SSE* values of the LRCs in the light-saturated (200 to 1000 μmol (photon) m^–2^ s^–1^) stage across HL-1 (**a**), HL-2 (**b**), and LL (**c**) and overall set (**d**). Red + indicate outliers, defined as values beyond the minimum and maximum values within 1.5 times the IQR from the first and third quartiles.

**Figure 8:**
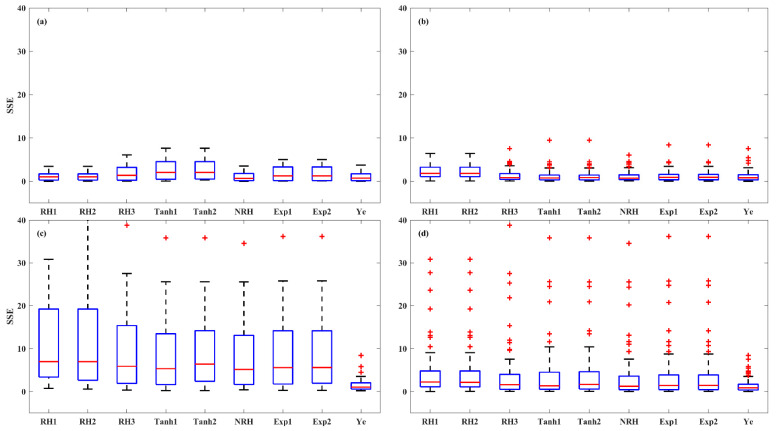
*SSE* values of the LRCs in the photoinhibition stage (1000 to 2000 μmol (photon) m^–2^ s^–1^) across HL-1 (**a**), HL-2 (**b**), and LL (**c**) and overall set (**d**). Red + indicate outliers, defined as values beyond the minimum and maximum values within 1.5 times the IQR from the first and third quartiles.

The accuracy of the models was further evaluated in three irradiance stages of LRC: light-limited (0 and 200 μmol (photon) m^–2^ s^–1^), light-saturated (200 to 1000 μmol (photon) m^–2^ s^–1^), and photoinhibition (1000 to 2000 μmol (photon) m^–2^ s^–1^) stages, across HL-1, HL-2, and LL and overall set (Figure 6, Figure 7 and Figure 8). At light-limited stage, the Ye (median *SSE* of 0.51) and NRH (median *SSE* of 0.59) model demonstrated superior performance, followed by RH3 (median *SSE* of 0.88), while the RH2 and RH1 performed worst (median *SSE* greater than 1.20). At the light-saturated stage, the performance of the Ye model demonstrated it was clearly superior to the other eight models, with a median *SSE* of 0.49, while the median *SSEs* for the other models ranged between 0.96 and 2.29. At the photoinhibition stage, the Ye model remained the best performance, with a median *SSE* of 0.87, while the median *SSEs* of other eight LRC models were greater than 1.20, with a large number of outliers observed. Specifically, in the light-saturated and photoinhibition stages of LL leaves, the Ye model was the best model, with a median *SSE* of 0.59 and 1.03, respectively, and the other eight models fell substantially behind the Ye model. Overall, the Ye model yielded the best results.

### 2.3. Comparisons of Photosynthetic Parameters

**Figure 9:**
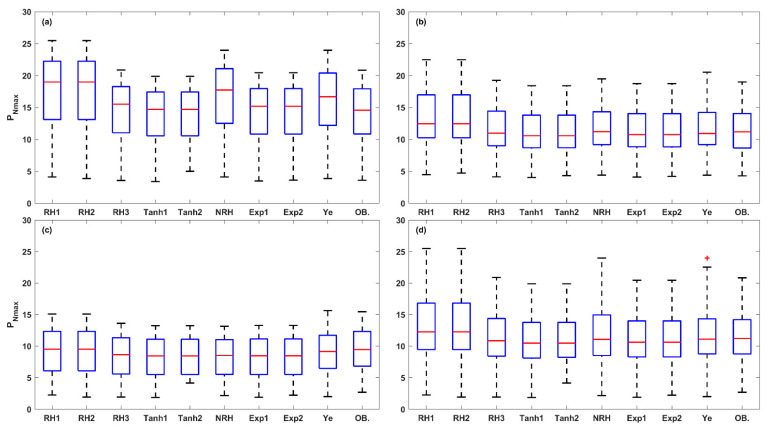
The box plots of *P_Nmax_* values estimated from the nine LRC models compared across HL-1 (**a**), HL-2 (**b**), and LL (**c**) and the overall set (**d**). (OB. denotes the observation of *P_Nmax_*). Red + indicate outliers, defined as values beyond the minimum and maximum values within 1.5 times the IQR from the first and third quartiles.

**Figure 10:**
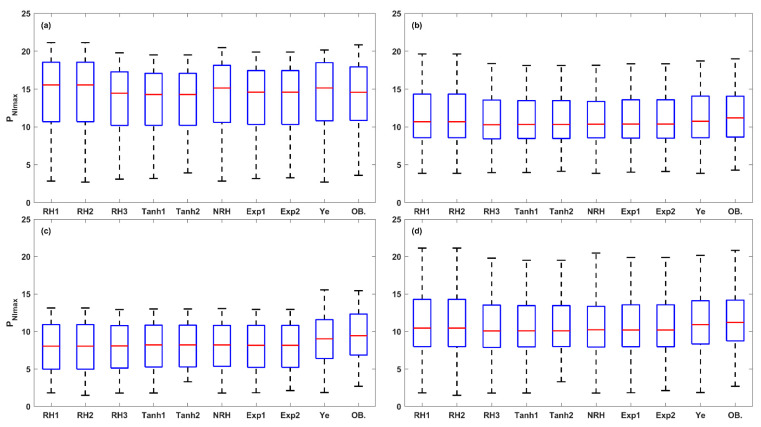
The box plots of *P_NImax_* values estimated from the nine LRC models compared across HL-1 (**a**), HL-2 (**b**), and LL (**c**) and the overall set (**d**) (OB. denotes the observation of *P_Nmax_*).

**Figure 11:**
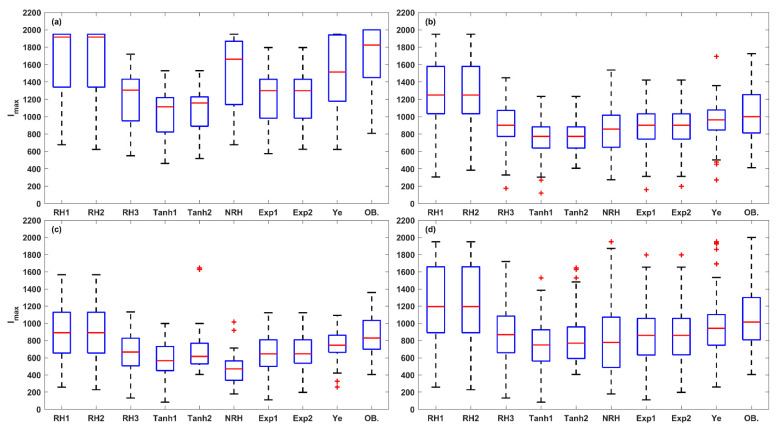
The box plots of *I_max_* values estimated from the nine LRC models compared across HL-1 (**a**), HL-2 (**b**), and LL (**c**) and the overall set (**d**) (OB. denotes the observation of *I_sat_*). Red + indicate outliers, defined as values beyond the minimum and maximum values within 1.5 times the IQR from the first and third quartiles.

**Figure 12:**
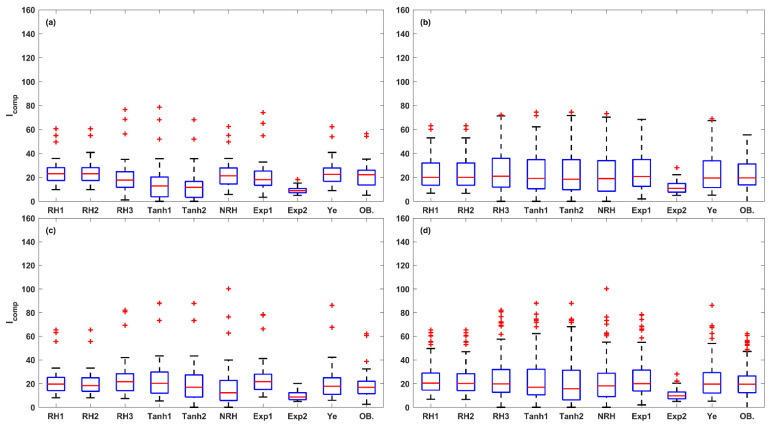
The box plots of *I_comp_* values estimated from the nine LRC models compared across HL-1 (**a**), HL-2 (**b**), and LL (**c**) and the overall set (**d**) (OB. denotes the observation of *I_comp_*). Red + indicate outliers, defined as values beyond the minimum and maximum values within 1.5 times the IQR from the first and third quartiles.

**Figure 13:**
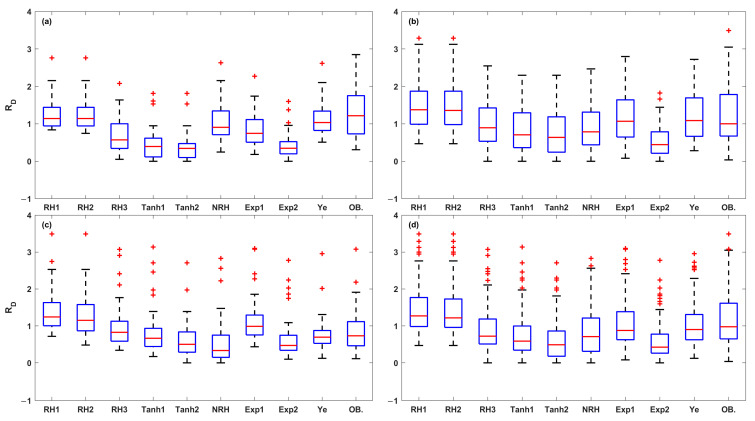
The box plots of *R_D_* values estimated from the nine LRC models compared across HL-1 (**a**), HL-2 (**b**), and LL (**c**) and the overall set (**d**) (OB. denotes the observation of *R_D_*). Red + indicate outliers, defined as values beyond the minimum and maximum values within 1.5 times the IQR from the first and third quartiles.

Further, a total of 15 photosynthetic parameters were obtained from the fitted LRCs. The box plot analysis in Figure 9, Figure 10, Figure 11, Figure 12 and Figure 13 compares the five key photosynthetic parameters (*P_Nmax_*, *P_NImax_*, *I_max_*, *R_D_*, and *I_comp_*) from the nine models in terms of light-acclimation type (HL-1, HL-2, and LL) and overall set. The other 10 photosynthetic parameters were detailed in the Supporting Materials (Figures S2–S11). The box plots were utilized to depict the distribution of the photosynthetic parameters by representing the minimum, median, and maximum values. Red + indicate outliers in the boxplots of Figure 9, Figure 10, Figure 11, Figure 12 and Figure 13 and Figures S2–S11.

The estimation of *P_Nmax_* reveals diverse results among the nine LRC models. Figure 9 presents a box plot analysis of the *P_Nmax_* values obtained from the models when applied to the three light-acclimation types (a–c) and the overall set (d). The results indicate that all LRC models overestimated the *P_Nmax_* in HL-1 leaves, particularly in the RH1, RH2, and NRH models, where the predicted *P_Nmax_* values were up to 20% greater than the observed values. In contrast, the *P_Nmax_* predictions from Tanh1, Tanh2, Exp1, and Exp2 models were relatively more accurate compared to the other models. For the *P_Nmax_* estimates in HL-2 leaves, the majority of models, except for RH1 and RH2, achieved good results, with a small difference in median values between predicted and observed values (−0.62 to 0.02 μmol (CO_2_) m^−2^ s^−1^). In LL leaves, all nine LRC models underestimated the *P_Nmax_*, with the estimates from the RH1, RH2, and Ye models being closest to the observations. Overall, the Ye model demonstrated the best performance for predicting *P_Nmax_* across the overall set, with a small difference in median values between the predicted and observed values (−0.10 μmol (CO_2_) m^−2^ s^−1^), followed by NRH, RH3, Exp1, Exp2, Tanh1, Tanh2, RH1, and RH2 models.

The estimations of *P_NImax_* from the nine models were compared to the observed *P_Nmax_* values, indicated as OB’ in Figure 10. The *P_NImax_* was found to be a good description of the *P_Nmax_* observations, with a difference in median values between predicted and observed values ranging from −1.40 to 0.97 μmol (CO_2_) m^−2^ s^−1^. The three models with the highest accuracy for estimating *P_NImax_* were identified as follows: the Exp1, Exp2, and RH3 models for HL-1; the Ye, RH1, and RH2 models for HL-2; and the Ye, Tanh1, and Tanh2 models for LL. Overall, the Ye model demonstrated the best performance for predicting *P_NImax_* across the overall set, with a small difference in median values between the predicted and observed values (−0.28 μmol (CO_2_) m^−2^ s^−1^), followed by RH1, RH2, NRH, Exp1, Exp2, Tanh1, Tanh2, and RH3 models.

The *I_max_* obtained from these models was also compared with the observed *I_sat_* values, revealing significant discrepancies (Figure 11). In HL-1 and LL leaves, the RH1 and RH2 models outperformed the other models. In the HL-2 and the overall set, the Ye model performed best, followed by the RH3, Exp1, and Exp2 models. The overall model performance, ranked from high to low, was as follows: Ye, RH3, Exp1, Exp2, RH2, RH1, NRH, Tanh2, and Tanh1 models.

With regards to estimating the *I_comp_* (Figure 12), the top three models in terms of accuracy were the Ye, RH2, and RH1 models in HL-1 and HL-2; and the Tanh2, Ye, and RH2 models in LL. The RH1 and RH2 models consistently overestimated *I_comp_*, while the NRH and Exp2 models consistently underestimated *I_comp_* across HL-1, HL-2, LL, and the overall set. Overall, the Ye model demonstrated the highest accuracy in the estimation of *I_comp_* across the overall set, with a small difference in median values between the predicted and observed values (0.11 μmol (photon) m^–2^ s^–1^). The overall model performance, ranked from high to low, was as follows: Ye, RH2, RH1, NRH, RH3, Exp1, Tanh2, Tanh1, and Exp2 models.

When it comes to fitting the *R_D_* (Figure 13), there are significant differences among the nine LRC models. The top three models in terms of accuracy within their respective types were the RH1, RH2, and Ye models for HL-1; the Exp1, Ye, and RH3 models for HL-2; and the Ye, Tanh1, and RH3 models for LL. The Tanh1, Tanh2, NRH, and Exp2 models underestimated the *R_D_* across HL-1, HL-2, LL, and the overall set. The RH1 and RH2 models yielded the best results in HL-1 leaves; however, they tended to overestimate *R_D_* in HL-2 and LL leaves. Overall, the Ye model yielded the most accurate estimates, with a small difference in median values between the predicted and observed values (−0.075 μmol (CO_2_) m^−2^ s^−1^). The model performance, ranked from high to low, was as follows: Ye, Exp1, RH2, RH3, NRH, RH1, Tanh1, Tanh2, and Exp2 models.

Additionally, the *I_sat_* cannot be obtained directly from the first eight models, except for the Ye model, as shown in Figure S2. In HL-2 and LL, the Ye model demonstrated relatively good performance in estimating the *I_sat_* observation, with median difference between the predicted and observed values of 287.18 and 112.37 μmol (photon) m^–2^ s^–1^, respectively. However, the Ye model exhibited an obvious overestimation of *I_sat_* in HL-1, with many values exceeding the normal range (values large than 2500 μmol (photon) m^–2^ s^–1^ were reset to 2500 μmol (photon) m^–2^ s^–1^ in the box plot). The *I_sat95_* calculated from the nine LRC models was the most consistent with the *I_sat_* observations compared to the *I_sat50_*, *I_sat85_*, *I_sat90_*, and *I_sat_* values (refer to Figures S3–S6). Notably, the RH3 in HL-2, LL, and the overall set, yielded the best *I_sat95_* predictions for estimating *I_sat_* observations, with median differences ranging from −47 to 48 μmol (photon) m^–2^ s^–1^, and second-best performance in HL-1. The Ye model performed best *I_sat95_* for estimating *I_sat_* observations in HL-1, although there were several significantly overestimated values. The overall model performance of *I_sat95_* for estimating *I_sat_* observations, ranked from high to low, was as follows: RH3, Ye, Exp1, Exp2, Tanh2, Tanh1, NRH, RH1, and RH2 models.

Comparing the *P_gmax_* values obtained from the nine models (Figure S7), the Exp1, RH3, and Exp2 models in HL-1; the RH3, Ye, and Exp1 models in HL-2; and the Ye, RH1, and RH2 models in LL were the top three performers compared to the other models. The RH1 and RH2 models overestimated *P_gmax_*, while the Tanh2, Tanh1, and Exp2 models underestimated *P_gmax_* across the HL-1, HL-2, LL, and the overall set. In the overall set, the Ye model yielded the most accurate estimates, with a small median difference of −0.04 μmol (CO_2_) m^−2^ s^−1^ between the predicted and observed values. The overall model performance, ranked from high to low, was as follows: Ye, RH3, Exp1, Tanh1, Tanh2, NRH, Exp2, RH1, and RH2 models.

The quantum yield of photosynthesis at different light intensities, including *ϕ_(I0)_*, *ϕ_(Icomp)_*, *ϕ_(I0_Icomp)_*, and *ϕ_(Icomp_I200)_*, was analyzed and presented in Figures S8–S11. The results indicated diverse median, minimum, and maximum values for these parameters. Overall, all nine LRC models predicted similar values for *ϕ_(Icomp_I200)_*, which were lower than those of *ϕ_(I0)_*, *ϕ_(Icomp)_*, and *ϕ_(I0_Icomp_*_)_. Across HL-1, HL-2, LL and the overall set, the RH1 and RH2 models predicted higher values for *ϕ_(I0)_*, *ϕ_(Icomp)_*, and *ϕ_(I0_Icomp)_* compared to the other models. In contrast, the RH3, Tanh1, Tanh2, and NRH models produced lower and similar values of *ϕ_(I0)_*, *ϕ_(Icomp)_*, and *ϕ_(I0_Icomp)_* than other models. The predicted values obtained from the Exp1, Exp2, and Ye models were median estimates that were comparable across all types and the overall set.

## 3. Data and Method

### 3.1. Experiment Design

Field experiments were conducted between 2015 and 2017 at the experimental zone of Shanghai Engineering Research Center of Low-Carbon Agriculture, located at the Zhuanghang experimental station of the Shanghai Academy of Agricultural Sciences in Shanghai, China (30°53′ N, 121°23′ E). The experimental station boasts a humid subtropical climate, characterized by an average annual temperature of approximately 16.6 °C and an average annual precipitation of 1353 mm. The plow layer of the soil is about 15 cm in depth, with a soil organic carbon concentration of 13.7 g kg^−1^, a total nitrogen concentration of 1.4 g kg^−1^, a total phosphorus concentration of 0.80 g kg^−1^, a soil bulk density of 1.4 g cm^−3^, and a pH of 7.6. The rice varieties Huayou14 (used in 2015 and 2016), Huhan61 and Xiushui134 (both used in 2017) were selected due to their distinct differences in canopy structure and leaf color.

(1) Nitrogen fertilizer experiment: From 2015 to 2017, four nitrogen fertilizer rate treatments were used: 0, 100, 200, and 300 kg hm^−2^, denoted as N0, N100, N200, and N300, respectively, and the rice varieties were Huayou14 in 2015 and 2016, and Huhan61 in 2007. The nitrogen fertilizer was split into three stages: 50% base fertilizer, 30% tiller fertilizer, and 20% heading fertilizer. Additionally, a phosphorus (P_2_O_5_) fertilizer of 100 kg hm^−2^ was applied once as a base fertilizer for each treatment, and a potassium (K_2_O) fertilizer of 225 kg hm^−2^ was used, with 44% applied as a base and 56% as a heading fertilizer.
(2) Irrigation experiment: In 2017, three irrigation treatments were applied at 100%, 50%, and 20% of the conventional irrigation rate, and the rice varieties were Huhan61 and Xiushui134, denoted as HH100, HH50, HH20, and XS100, XS50, XS20, respectively. The nitrogen fertilizer rate used in the irrigation experiment was consistent with the N200 level (200 kg hm^−2^), with the same phosphorus and potassium fertilizers as those used in the nitrogen fertilizer experiments.

The nitrogen fertilizer and irrigation treatments were arranged using a randomized block design and replicated three times. Each experimental plot had an area of 56 m^2^ (7 m × 8 m), and other management practices were consistent with those used in conventional high-yield fields.

### 3.2. Photosynthesis Measurement

LRCs were measured in situ for rice grown in the field using a portable photosynthetic system (LI-6400, manufactured by LI-COR, Inc. in Lincoln, NE, USA) coupled with a red-blue LED light source (6400-02B, Li-Cor). An auto-program was used to collect data at 13 irradiance levels (2000, 1600, 1300, 1000, 800, 600, 400, 200, 150, 100, 50, 25, 0 µmol m^−2^ s^−1^), beginning at the 2000 µmol m^−2^ s^−1^ light level and allowing up to 3 min of acclimation at each level before data-logging. The reference CO_2_ concentration was set to 400 ppm, and the block temperature was maintained at ambient conditions (ranging from 24 °C to 45 °C). Leaf widths were measured to correct gas exchange data for the actual leaf area.

The measurements were conducted between 10:00 to 16:00 on clear days shortly after the flag leaf fully emerged, approximately from late August to early November, during 2015–2017. The flag leaf (FL) and the next two leaves 2L, 3L were measured in the nitrogen fertilizer experiment, while only the FL and 2L were measured in the irrigation experiment. A total of 108 LRCs were measured from rice leaves, covering three varieties, four nitrogen levels, three irrigation levels, three leaf positions, and various measurement dates (leaf ages). These details are summarized in Table S1.

### 3.3. LRC Model Fitting

The nine LRC models, including three rectangular hyperbola (RH) models (Equation (1) [15], Equation (2) [17], and Equation (3) [16]), two hyperbolic tangent (Tanh) models (Equation (4) [32] and Equation (5) [19]), one nonrectangular hyperbola (NRH) model (Equation (6) [20]), two exponential (Exp) models (Equation (7) [22], Equation (8) [23]), and one Ye model (Equation (9)) [13], were applied to fit the LRCs. The expressions and parameters of these models were as follows:

(1) $$P_{N}=\frac{\phi_{\left(I_{0}\right)}\times I\times P_{g\max}}{\phi_{\left(I_{0}\right)}\times I+P_{g\max}}-R_{D}$$

(2) $$P_{N}=\frac{I\times P_{g\max}}{I+I_{\left(50\right)}}-R_{D}$$

(3) $$P_{N}=\frac{\phi_{\left(I_{0}\right)}\times I\times P_{g\max}}{\sqrt{{\phi_{\left(I_{0}\right)}}^{2}\times I^{2}+{P_{g\max}}^{2}}}-R_{D}$$

(4) $$P_{N}=P_{g\max}\times \tanh(\frac{\phi_{\left(I_{0}\right)}\times I}{P_{g\max}})-R_{D}$$

(5) $$P_{N}=P_{g\max}\times \tanh(\frac{I}{I_{sat}})-R_{D}$$

(6) $$P_{N}=\frac{\phi_{\left(I_{0}\right)}\times I+P_{g\max}-\sqrt{{\left(\phi_{\left(I_{0}\right)}\times I+P_{g\max}\right)}^{2}-4\theta \times \phi_{\left(I_{0}\right)}\times I\times P_{g\max}}}{2\theta }-R_{D}$$

(7) $$P_{N}=P_{g\max}\times (1-\exp(\frac{-\phi_{\left(I_{0}\right)}\times I}{P_{g\max}}))-R_{D}$$

(8) $$P_{N}=P_{g\max}\times (1-\exp(-k(I-I_{comp})))-R_{D}$$

(9) $$P_{N}=\phi_{\left(I_{0}-I_{comp}\right)}\times \frac{1-\beta \times I}{1+\gamma \times I}\times (I-I_{comp})$$

where *I* is the photosynthetic photon flux density (μmol (photon) m^−2^ s^−1^), *I_comp_* is the light-compensation point (μmol (photon) m^−2^ s^−1^), *I_sat_* is the light-saturation point (μmol (photon) m^−2^ s^−1^), *I_(50)_* is the light-saturation point at 50% of *P_gmax_* (μmol (photon) m^−2^ s^−1^), *P_N_* is the net photosynthetic rate (μmol (CO_2_) m^−2^ s^−1^), *P_gmax_* is the maximum gross photosynthetic rate (μmol (CO_2_) m^−2^ s^−1^), *R_D_* is the dark respiration rate (μmol (CO_2_) m^−2^ s^−1^). The quantum yield of photosynthesis (*ϕ*) is a measure of photosynthetic efficiency expressed in moles of photons absorbed per mole of CO_2_ fixed. Specifically, *ϕ_(I0_*_)_ is the quantum yield at *I* = 0 (μmol (CO_2_) μmol (photon)^−1^), which is calculated as the derivative of LRC model at *I* = 0 μmol (photon) m^−2^ s^−1^, and *ϕ_(I0-Icomp)_* is the quantum yield with I between *I_0_* and *I_comp_* (μmol (CO_2_) μmol (photon)^−1^). *θ* and *k* define the convexity, shaping the function, while *β* and *γ* are the photoinhibition coefficient and saturation coefficients, respectively. The LRC model fitting was completed using the nonlinear least-squares regression function in MATLAB 2016a. 

### 3.4. Photosynthetic Parameters

The 15 photosynthetic parameters can be obtained through LRC model fitting, as listed in Table 1. For the detailed calculation procedures, refer to the publication by Lobo et al. (2013) [14]. Additionally, observations of the four key photosynthetic parameters, including the maximum net photosynthetic rate (*P_Nmax__ob*), light-saturation point (*I_sat__ob*), light-compensation point (*I_comp__ob*), and dark respiration rate (*R_D__ob*), can be approximately obtained directly from the LRC observations, with detailed calculations presented in Table 1.

### 3.5. Model Comparison Criterion

The performance of the nine LRC models was evaluated and compared using the coefficient of determination (*R^2^*), the sum of squared errors (*SSE*), and the Akaike information criterion (*AIC*). *R^2^* provides a measure of how well the regression model fits the observed data, expressing the proportion of variability in the observations that can be explained by the model. *SSE* represents the sum of the squared differences between each observation and its corresponding fitting value. *AIC* is used to determine which model best explains the variance in the *P_N_* with the fewest estimated parameters. A smaller *AIC* value indicates a better fit of the model to the data. Additionally, the *SSE* was calculated for three irradiance stages in each LRC: In the light-limited stage, where *I* between 0 and 200 μmol (photon) m^–2^ s^–1^, a linear response of *P_N_* to *I* can be seen. In the light-saturated stage, as *I* increases from 200 to 1000 μmol (photon) m^–2^ s^–1^, the increase in *P_N_* slows down and curvilinearly approaches or reaches saturation. In the photoinhibition stage, as *I* increases further from 1000 to 2000 μmol (photon) m^–2^ s^–1^, there may be a decline in *P_N_* resulting from photoinhibition. The equations of *R^2^*, *SSE*, and *AIC* are provided below:

(10) $$R^{2}=1-\frac{\sum_{i=1}^{n}{\left(y_{i}-y_{i}^{\prime }\right)}^{2}}{\sum_{i=1}^{n}{\left(y_{i}-{\bar{y}}_{i}\right)}^{2}}$$

(11) $$SSE=\sum_{i=1}^{n}{\left(y_{i}-y_{i}^{\prime }\right)}^{2}$$

(12) $$AIC=2k+nln\frac{\sum_{i=1}^{n}{\left(y_{i}-y_{i}^{\prime }\right)}^{2}}{n}$$

where $y_{i}$, $y_{i}^{\prime }$, and ${\bar{y}}_{i}$ are the observations, the model predicted values, and the mean of the observations, respectively. *n* is the number of observations in one LRC (*n* = 13 in this study). *k* is the number of estimated regression parameters.

## 4. Discussion

In this study, the performance of model accuracy was compared using our newly constructed LRC set. Unlike previous research that was based on a limited leaf traits and a small number of LRC observations [12,28,29,30], we measured 108 LRCs in situ for rice grown in the field across 3 years under different leaf positions, leaf ages (measurement dates), nitrogen levels, irrigation levels, and varieties, producing diverse phenotypic variations in leaf photosynthetic traits.

Photosynthetic acclimation made the leaf to optimize light utilization and protect against the potential stress from excess light. Our results demonstrate that the measured rice leaf grow in field showed diversity in photosynthetic acclimation, causing the shape of LRCs varies significantly under a range of leaf traits, which can be specifically categorized into HL-1, HL-2, and LL leaves (Figure 1). HL-1 leaves mostly observed at the top of the canopy (FL and 2L) with N200 and N300 levels, which are exposed to high-light conditions. In contrast, LL leaves mainly occur at the base part of the canopy (3L) with N300 level, which are adapted to low-light conditions. The HL-2 leaves are typically observed at the lower of the canopy (2L) with N200 level, which are exposed to high-light conditions. The complexity of the LRCs under varying irradiance levels is further compounded by leaf ages and water availability.

A robust LRC model should accurately reproduce the leaf-scale *P_N_* responses across all light stages, including the light-limited (0–200 μmol (photon) m^−2^ s^−1^), light-saturated (200–1000 μmol (photon) m^−2^ s^−1^), and photoinhibition stages (1000–2000 μmol (photon) m^−2^ s^−1^) [12]. The LRCs are usually fitted and evaluated using all irradiance levels, ranging from 0 to 2000 μmol (photon) m^−2^ s^−1^ [12,13,14]. Our finding indicates that the accuracy of LRC models varies significantly across the three stages, particularly in the photoinhibition stage. In the photoinhibition stage of LL leaves, the Ye model was the best model (a median *SSE* of 1.03), and the other eight models fell substantially behind (median *SSE* larger than 5.16) (Figure 8). The Ye model is a mechanistic model specifically developed to describe the light-harvesting characteristics and biophysical parameters of photosynthetic pigment molecules, grounded in the principles of photosynthetic electron transport through photosystem II (PSII). The Ye model replaces *P_Nmax_* in the RH model with *β* and *γ*, resulting a non-asymptotic function. Both *β* and *γ* have clearly biophysical meanings: *β* quantifies the degree of dynamic downregulation of PSII, while *γ* serves as a saturation term describing the response of photosynthetic electron transport rate to light intensity [33,34]. Therefore, the Ye model is suitable for complex LRC shapes, particularly in light-saturation and photoinhibition stages, aligning with previous research [12,13,14].

The RH, Tanh, NRH, and Exp models are asymptotic function that cannot directly calculate *P_Nmax_* and *I_sat_*. Lobo et al. (2013) proposed using *I_max_* instead of *I_sat_*, defining it as the point beyond which there is no significant change in *P_N_* [14]. Our results indicate that the *I_max_* is superior to *I_sat(n)_* for estimating *I_sat_* observations, and the *I_max_* and *P_NImax_* values from Ye model have the highest accuracy, being closer to the observed values of *I_sat_* and *P_Nmax_*.

While the study provided valuable insights, there are some limitations that need to be acknowledged. Some photosynthetic parameters that were not directly measured, such as *ϕ_(I0)_*, *ϕ_(Icomp)_*, *ϕ_(I0_Icomp)_*, and *ϕ_(Icomp_I200)_*, were only evaluated based on the overall model fitting accuracy and parameter differences between models. In addition, this study was limited to observing photosynthesis at the leaf scale, and as a result, the model comparison results are only applicable to LRCs at that scale. Future research should aim to investigate photosynthesis at the canopy scale to better understand photosynthetic responses in field and plant responses to environmental conditions [35,36]. Additionally, while empirical and mechanistic models remain valuable in current research due to their simplicity, and parameters calculated from these models effectively describe the photosynthetic capacity, efficiency, and other related traits [11,33,34], advanced computational approaches, such as artificial intelligence, have the potential to reveal complex, non-linear patterns in LRCs, presenting promising directions for future research.

## 5. Conclusions

The performance of nine well-known LRC models was compared using 108 LRCs in situ for rice grown in the field across 3 years under different leaf positions, leaf ages (measurement dates), nitrogen levels, irrigation levels, and varieties. This comparison was conducted by classifying the varying shapes of LRCs into three light-acclimation types (HL-1, HL-2, and LL leaves) and further analyzing them across the three irradiance stages of each LRC: light-limited, light-saturated, and photoinhibition. The Ye model was chosen as the optimal model for fitting leaf LRCs in rice, not only due to its strong performance in model fitting but also for its success in accurately predicting key photosynthetic parameters (*P_Nmax_*, *I_sat_*, *R_D_*, and *I_comp_*). *P_NImax_* and *I_max_* were the most appropriate parameters to describe photosynthetic capacity at the light-saturation point. This study not only helps to explore the best model for quantifying the LRCs but also provides a scientific and effective guidance for accurately estimating the photosynthetic parameters.

## Figures and Tables

**Table 1:** Calculation methods for photosynthetic parameters derived from LRC models or observational measurements.

Parameter	Description
*I_comp_*	The light-compensation point was either a regression parameter in Equations (8) and (9) or calculated by finding the value of *I* in the model when *P_N_* = 0.
*I_sat_*	The light-saturation point is the value of *I* when *P_N_* = *P_Nmax_*_._
*I_max_*	The maximum value of *I* was defined as the point at which an increase in *I* no longer leads to a significant increase in *P_N_*.
*I_sat(n)_*	The light-saturation points at a specific percentile (*n*) of *P_Nmax_*. *I_sat95_*, *I_sat90_*, *I_sat85_*, and *I_sat50_* were the light-saturation point for *P_N_* + *R_D_* equal to 95%, 90%, 85%, 50% of *P_Nmax_*, respectively.
*P_gmax_*	The maximum gross photosynthetic rate was either a regression parameter from Equations (1)–(8) or estimated as *P_Nmax_* + *R_D_* in Equation (9).
*P_Nmax_*	The maximum net photosynthetic rate was estimated using *P_gmax_*–*R_D_* in Equations (1)–(8), or calculated as *P_N_* at *I_sat_* in Equation (9).
*P_NImax_*	The net photosynthetic rate at *I_max_*.
*R_D_*	The dark-respiration rate was either a regression parameter in Equations (1)–(8) or estimated *P_N_* at *I* = 0 in Equation (9).
*ϕ_(I0_*_)_	The quantum yield at *I* = 0 was calculated as the derivative of LRC model at *I* = 0 μmol (photon) m^−2^ s^−1^.
*ϕ_(Icomp_*_)_	The quantum yield at *I* = *I_comp_* was calculated as the derivative of the LRC model at *I* = *I_comp_* μmol (photon) m^−2^ s^−1^.
*ϕ_(I0_*__*Icomp*)_	The quantum yield in the range of *I* between *I_0_* and *I_comp_* was calculated as the slope of the linear regression of *P_N_* over the range of *I* between 0 and *I_comp_* μmol (photon) m^−2^ s^−1^.
*ϕ_(Icomp_*__*I200*)_	The quantum yield in the range of *I* between *I_comp_* and *I_200_* was calculated as the slope of the linear regression of *P_N_* and *I* over the range of *I* between *I_comp_* and 200 μmol (photon) m^−2^ s^−1^.
*P_Nmax__ob*	The observed maximum net photosynthetic rate.
*I_sat__ob*	The observed light-saturation points.
*I_comp__ob*	The observed light-compensation point was calculated as the slope of linear regression of *P_N_* at 0, 25 and 50 μmol (photon) m^−2^ s^−1^.
*R_D__ob*	The observed dark-respiration rate was *P_N_* at *I* = 0.

## Data Availability

The data presented in this study are available upon request from the corresponding author.

## Supplementary Materials

The following supporting information can be downloaded at: https://www.mdpi.com/article/10.3390/plants14010023/s1, Text S1: A detailed statistical analysis of the three leaf types in relation to the leaf positions; Table S1: Measurement dates, varieties, treatments, and leaf positions for LRCs collected in the study; Figure S1: Statistics of HL-1, HL-2, and LL leaves under different nitrogen levels and leaf positions; a: all leaves under different light-acclimation types (HL-1, HL-2, and LL) across four nitrogen levels (N0, N100, N200, and N300); b, c, and d represent the HL-1, HL-2, and LL leaves, respectively, at different leaf positions (FL, 2L, and 3L) across four nitrogen levels (N0, N100, N200, and N300); Figure S2: The box plots of *I_sat_* values estimated from the nine LRC models compared across HL-1 (a), HL-2 (b), and LL (c) and the overall set (d) (OB. denotes the observation of *I_sat_*); Figure S3: The box plots of *I_sat50_* values estimated from the nine LRC models compared across HL-1 (a), HL-2 (b), and LL (c) and the overall set (d) (OB. denotes the observation of *I_sat_*); Figure S4: The box plots of *I_sat85_* values estimated from the nine LRC models compared across HL-1 (a), HL-2 (b), and LL (c) and the overall set (d) (OB. denotes the observation of *I_sat_*); Figure S5: The box plots of *I_sat90_* values estimated from the nine LRC models compared across HL-1 (a), HL-2 (b), and LL (c) and the overall set (d) (OB. denotes the observation of *I_sat_*); Figure S6: The box plots of *I_sat95_* values estimated from the nine LRC models compared across HL-1 (a), HL-2 (b), and LL (c) and the overall set (d) (OB. denotes the observation of *I_sat_*); Figure S7: The box plots of *P_gmax_* values estimated from the nine LRC models compared across HL-1 (a), HL-2 (b), and LL (c) and the overall set (d), and compared with the observations of *P_gmax_* (OB.); Figure S8: The box plots of *ϕ_(I0)_* values estimated from the nine LRC models compared across HL-1 (a), HL-2 (b), and LL (c) and the overall set (d); Figure S9: The box plots of *ϕ_(Icomp)_* values estimated from the nine LRC models compared across HL-1 (a), HL-2 (b), and LL (c) and the overall set (d); Figure S10: The box plots of *ϕ_(I0_Icomp)_* values estimated from the nine LRC models compared across HL-1 (a), HL-2 (b), and LL (c) and the overall set (d); Figure S11: The box plots of *ϕ_(Icomp_I200)_* values estimated from the nine LRC models compared across HL-1 (a), HL-2 (b), and LL (c) and the overall set (d).

## References

[1] Evans J.R. (2013). Improving photosynthesis. Plant Physiol..

[2] Long Stephen P., Marshall-Colon A., Zhu X.-G. (2015). Meeting the Global Food Demand of the Future by Engineering Crop Photosynthesis and Yield Potential. Cell.

[3] Ort D.R., Merchant S.S., Alric J., Barkan A., Blankenship R.E., Bock R., Croce R., Hanson M.R., Hibberd J.M., Long S.P. (2015). Redesigning photosynthesis to sustainably meet global food and bioenergy demand. Proc. Natl. Acad. Sci. USA.

[4] Zhu X.-G., Long S.P., Ort D.R. (2010). Improving photosynthetic efficiency for greater Yield. Annu. Rev. Plant Biol..

[5] Smith E.N., van Aalst M., Tosens T., Niinemets Ü., Stich B., Morosinotto T., Alboresi A., Erb T.J., Gómez-Coronado P.A., Tolleter D. (2023). Improving photosynthetic efficiency toward food security: Strategies, advances, and perspectives. Mol. Plant.

[6] Murchie E.H., Hubbart S., Chen Y., Peng S., Horton P. (2002). Acclimation of rice photosynthesis to irradiance under field conditions. Plant Physiol..

[7] Kull O. (2002). Acclimation of photosynthesis in canopies: Models and limitations. Oecologia.

[8] Luo X., Keenan T.F. (2020). Global evidence for the acclimation of ecosystem photosynthesis to light. Nat. Ecol. Evol..

[9] Walters R.G. (2005). Towards an understanding of photosynthetic acclimation. J. Exp. Bot..

[10] An S., Liu X., Wen B., Li X., Qi P., Zhang K. (2020). Comparison of the photosynthetic capacity of phragmites australis in five habitats in saline–alkaline wetlands. Plants.

[11] Ögren E., Evans J.R. (1993). Photosynthetic light-response curves: I. The influence of CO_2_ partial pressure and leaf inversion. Planta.

[12] Ye Z.P., Duan S.H., Chen X.M., Duan H.L., Gao C.P., Kang H.J., An T., Zhou S.X. (2021). Quantifying light response of photosynthesis: Addressing the long-standing limitations of non-rectangular hyperbolic model. Photosynthetica.

[13] Ye Z.-P. (2007). A new model for relationship between irradiance and the rate of photosynthesis in Oryza sativa. Photosynthetica.

[14] Lobo F.d.A., de Barros M.P., Dalmagro H.J., Dalmolin Â.C., Pereira W.E., de Souza É.C., Vourlitis G.L., Rodríguez Ortíz C.E. (2013). Fitting net photosynthetic light-response curves with Microsoft Excel—A critical look at the models. Photosynthetica.

[15] Baly E.C.C. (1935). The kinetics of photosynthesis. Proc. R. Soc. Lond. Ser. B Biol. Sci..

[16] Smith E.L. (1936). Photosynthesis in relation to light and carbon dioxide. Proc. Natl. Acad. Sci. USA.

[17] Kaipiainen E.L. (2009). Parameters of photosynthesis light curve in Salix dasyclados and their changes during the growth season. Russ. J. Plant Physiol..

[18] Platt T., Jassby A.D. (1976). The relationship between photosynthesis and light for natural assemblages of coastal marine phytoplankton. J. Phycol..

[19] Abe M., Yokota K., Kurashima A., Maegawa M. (2009). High water temperature tolerance in photosynthetic activity of Zostera japonica Ascherson & Graebner seedlings from Ago Bay, Mie Prefecture, central Japan. Fish. Sci..

[20] Prioul J.L., Chartier P. (1977). Partitioning of transfer and carboxylation components of intracellular resistance to photosynthetic CO_2_ fixation: A critical analysis of the methods used. Ann. Bot..

[21] Thornley J.H.M. (1998). Dynamic model of leaf photosynthesis with acclimation to light and nitrogen. Ann. Bot..

[22] Webb W.L., Newton M., Starr D. (1974). Carbon dioxide exchange of Alnus rubra. Oecologia.

[23] Prado C.H.B.A., De Moraes J.A.P.V. (1997). Photosynthetic capacity and specific leaf mass in twenty woody species of Cerrado vegetation under field conditions. Photosynthetica.

[24] Reavis M., Purcell L.C., Pereira A., Naithani K. (2023). Effects of measurement methods and growing conditions on phenotypic expression of photosynthesis in seven diverse rice genotypes. Front. Plant Sci..

[25] Chen L., Luo W., Huang J., Peng S., Xiong D. (2021). Leaf photosynthetic plasticity does not predict biomass responses to growth irradiance in rice. Physiol. Plant..

[26] Gu J., Zhou Z., Li Z., Chen Y., Wang Z., Zhang H., Yang J. (2017). Photosynthetic properties and potentials for improvement of photosynthesis in pale green leaf rice under high light conditions. Front. Plant Sci..

[27] Chen Z.Y., Peng Z.S., Yang J., Chen W.Y., Ou-Yang Z.M. (2011). A mathematical model for describing light-response curves in *Nicotiana tabacum* L.. Photosynthetica.

[28] Ye Z.-P., Yu Q. (2018). Comparison of new and several classical models of photosynthesis in response to irradiance. Chin. J. Plant Ecol..

[29] Song D., Ma L., Wang Q. (2021). Photosynthetic light response models for spring wheat mulching treatments in a northwestern China desert oasis. Crop Sci..

[30] Zhu T., Li J., Liu Y., Tong X., Yu Q. (2020). Leaf photosynthetic light response of summer maize: Comparison of models and analysis of parameters. Photosynthetica.

[31] Archontoulis S.V., Miguez F.E. (2015). Nonlinear regression models and applications in agricultural research. Agron. J..

[32] Jassby A.D., Platt T. (1976). Mathematical formulation of the relationship between photosynthesis and light for phytoplankton. L&O.

[33] Ye Z.-P., Robakowski P., Suggett D.J. (2013). A mechanistic model for the light response of photosynthetic electron transport rate based on light harvesting properties of photosynthetic pigment molecules. Planta.

[34] Ye Z.-P., Suggett D.J., Robakowski P., Kang H.-J. (2013). A mechanistic model for the photosynthesis–light response based on the photosynthetic electron transport of photosystem II in C3 and C4 species. New Phytologist..

[35] Kull O., Kruijt B. (1998). Leaf photosynthetic light response: A mechanistic model for scaling photosynthesis to leaves and canopies. Funct. Ecol..

[36] Meacham-Hensold K., Fu P., Wu J., Serbin S., Montes C.M., Ainsworth E., Guan K., Dracup E., Pederson T., Driever S. (2020). Plot-level rapid screening for photosynthetic parameters using proximal hyperspectral imaging. J. Exp. Bot..

