# Supplementary material for: Evaluating Photosynthetic Light Response Models for Leaf Photosynthetic Traits in Paddy Rice (Oryza sativa L.) Under Field Conditions
Source: Plants (Basel). 2024 Dec 25;14(1):23. doi: 10.3390/plants14010023 (PMC11723129; doi:10.3390/plants14010023)
Supplement: Supplementary file 1 [file plants-14-00023-s001.zip › plants-3258749-supplementary.pdf]

Supplementary Materials

Title: Evaluating Photosynthetic Light Response Models for Leaf Photosynthetic Traits in Paddy Rice (*Oryza sativa* L.) under Field Conditions

Xinfeng Yao [1,2], Huifeng Sun [3,4,5], Sheng Zhou[3,4,5] and Linyi Li [1,2,*]

[1] Institute of Agricultural Science and Technology Information, Shanghai Academy of Agricultural Sciences, Shanghai 201403, China; xinfengyao@saas.sh.cn (X.Y.)

[2] Key Laboratory of Intelligent Agricultural Technology (Yangtze River Delta), Ministry of Agriculture and Rural Affairs, Shanghai 201403, China;

[3] Eco-Environmental Protection Research Institute, Shanghai Academy of Agricultural Sciences, Shanghai 201403, China; sunhuifeng@saas.sh.cn (H.S.); zhous@outlook.com (S.Z.);

[4] Shanghai Engineering Research Centre of Low-Carbon Agriculture (SERCLA), Shanghai 201415, China;

[5] Key Laboratory of Low-Carbon Green Agriculture in Southeastern China, Ministry of Agriculture and Rural Affairs, Shanghai 201403, China.

* Correspondence: lly@saas.sh.cn.

Text S1: A detailed statistical analysis of the three leaf types in relation to the leaf positions

Table S1. Measurement dates, varieties, treatments, and leaf positions for LRCs collected in the study.

Figures S1–S11:

Figure S1: Statistics of HL-1, HL-2, and LL leaves under different nitrogen levels and leaf positions; a: all leaves under different light acclimation types (HL-1, HL-2, and LL) across four nitrogen levels (N0, N100, N200, and N300); b, c, and d represent the HL-1, HL-2,

and LL leaves, respectively, at different leaf positions (FL, 2L, and 3L) across four nitrogen levels (N0, N100, N200, and N300).

Figure S2: The boxplots of I_{sat} values estimated from the nine LRC models compared across HL-1 (a), HL-2 (b), and LL (c) and the overall set (d) (OB. denotes the observation of I_{sat}).

Figure S3: The boxplots of I_{sat50} values estimated from the nine LRC models compared across HL-1 (a), HL-2 (b), and LL (c) and the overall set (d) (OB. denotes the observation of I_{sat}).

Figure S4: The boxplots of I_{sat85} values estimated from the nine LRC models compared across HL-1 (a), HL-2 (b), and LL (c) and the overall set (d) (OB. denotes the observation of I_{sat}).

Figure S5: The boxplots of I_{sat90} values estimated from the nine LRC models compared across HL-1 (a), HL-2 (b), and LL (c) and the overall set (d) (OB. denotes the observation of I_{sat}).

Figure S6: The boxplots of I_{sat95} values estimated from the nine LRC models compared across HL-1 (a), HL-2 (b), and LL (c) and the overall set (d) (OB. denotes the observation of I_{sat}).

Figure S7: The boxplots of P_{gmax} values estimated from the nine LRC models compared across HL-1 (a), HL-2 (b), and LL (c) and the overall set (d), and compared with the observations of P_{gmax} (OB.).

Figure S8: The boxplots of $\phi_{(I0)}$ values estimated from the nine LRC models compared across HL-1 (a), HL-2 (b), and LL (c) and the overall set (d).

Figure S9: The boxplots of $\phi_{(Icomp)}$ values estimated from the nine LRC models compared across HL-1 (a), HL-2 (b), and LL (c) and the overall set (d).

Figure S10: The boxplots of $\phi_{(I0\_Icomp)}$ values estimated from the nine LRC models compared across HL-1 (a), HL-2 (b), and LL (c) and the overall set (d).

Figure S11: The boxplots of $\phi_{(Icomp\_I200)}$ values estimated from the nine LRC models compared across HL-1 (a), HL-2 (b), and LL (c) and the overall set (d).

Text S1:

A statistical analysis of HL-1 leaves in relation to the leaf positions and nitrogen levels was conducted, as shown in Figure S1b. A total of 24 HL-1 leaves was observed in 108 LRC observations. The 11 HL-1 leaves under N200 had the highest proportion compared to other nitrogen levels, with 63.6% primarily distributed in the FL leaf position. Overall, HL-1 leaves under different nitrogen levels were mainly found in the FL (50.0%) and 2L (37.5%) leaf positions, with only 3 leaves in the 3L position (12.5%). The HL-1 leaves were observed at different observation dates across the three rice varieties in this study, with proportions of 19.2%, 22.0%, and 50.0% for Huayou14, Huhan61, and Xiushui134, respectively.

The HL-2 type consists of 50 leaves involving different nitrogen levels and leaf positions. The highest number of leaves was observed under the N200 level, with 20 leaves mainly distributed in the 2L leaf position, accounting for 55.0%. Overall, the 2L leaf position had the highest proportion of HL-2 leaves (42.0%), followed by the FL position (34.0%) and the 3L position (24.0%). The HL-2 leaves appeared across different rice varieties, with proportions of 42.3%, 50.0%, and 50.0% for Huayou14, Huhan61, and Xiushui134, respectively.

The LL type comprises a total of 34 leaves. The highest number of LL leaves was found under the N300 level, with a total of 13 leaves, of which the majority (53.8%) were observed in the 3L position. LL leaves were observed only in Huayou14 and Huhan61, with proportions of 38.5% and 28.0%, respectively. LL leaves were mainly observed at later stages (relatively older leaf age).

Furthermore, for the LRCs under different irrigation levels, only HL-1 and HL-2 leaves were observed across the three irrigation levels. Leaves from HL-1 and HL-2 had similar proportions among the samples in the irrigation experiment, at 41.7% and 58.3%, respectively. A high proportion of 80% HL-1 leaves was observed in the FL position, while 71.4% of HL-2 leaves were found in the 2L position.

Table S1. Measurement dates, varieties, treatments, and leaf positions for LRCs collected in the study.

**Measurement date (Leaf age)**	**Variety**	**Treatment**	**Leaf position**
20150909	Huayou14	N000	FL,3L
		N200	FL,2L,3L
		N300	FL,2L,3L
		N000	FL,2L
20151002	Huayou14	N000	FL,2L
		N100	FL,2L,3L
		N200	FL,2L,3L
		N300	FL,2L,3L
		N200	FL
20160912	Huayou14	N000	FL,2L,3L
		N100	FL,2L,3L
		N200	FL,2L,3L
		N300	FL,2L

20160920	Huayou14	N000	FL,2L,3L
		N100	FL,2L,3L
		N200	FL,2L,3L
		N300	FL,2L,3L
20161024	Huayou14	N000	FL,2L,3L
		N100	FL,2L,3L
20161103	Huayou14	N300	FL,2L,3L
20170823	Huhan61	N000	FL,2L
		N100	FL,2L,3L
		N200	FL,2L,3L
		N300	FL,2L,3L
20170828	Huhan61	N200	FL,2L,3L
		N300	FL,2L,3L
		N200	FL,2L
20170913	Huhan61	N000	FL,2L,3L
		N100	FL,2L
		N200	FL,2L,3L
		N300	FL,2L,3L
20171007	Huhan61	N000	FL,2L,3L
		N100	FL,2L,3L
		N200	FL,2L,3L
		N300	FL,2L,3L
20170912	Huhan61	HH20	FL,2L

	HH50	FL,2L
	HH100	FL,2L
Xiushui134	XS20	FL,2L
	XS50	FL,2L
	XS100	FL,2L

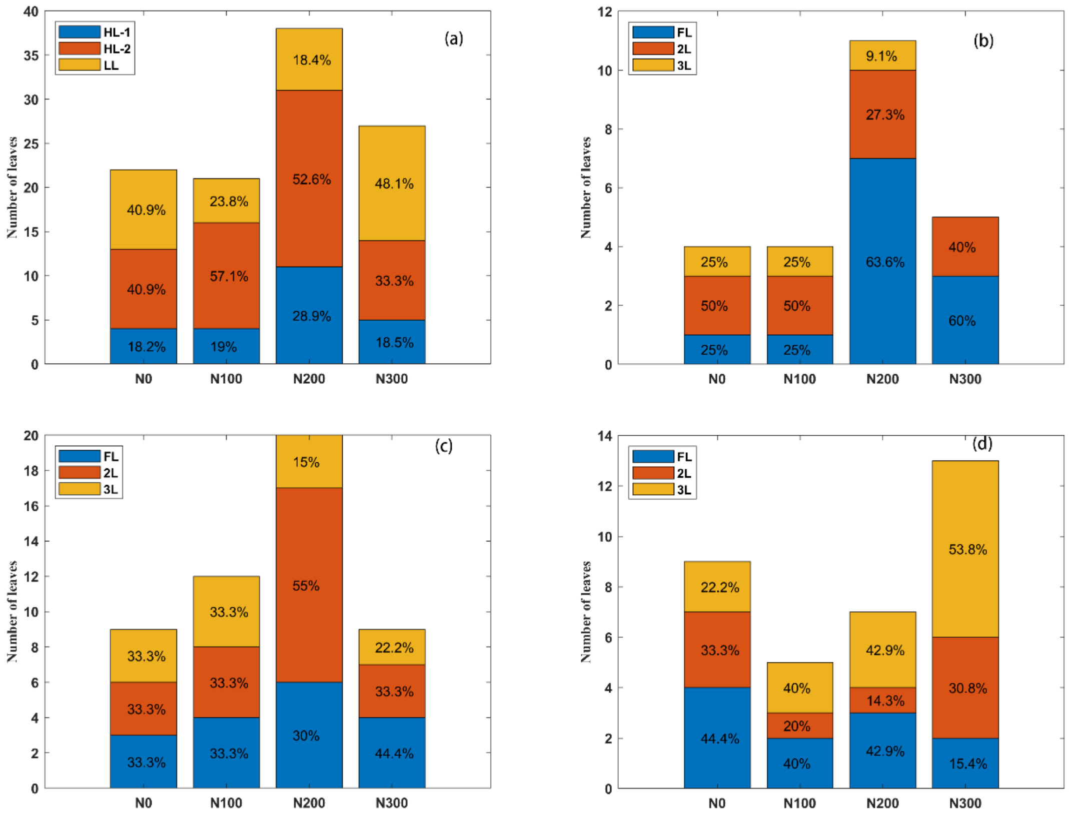

Figure S1: Statistics of HL-1, HL-2, and LL leaves under different nitrogen levels and leaf positions; a: all leaves under different light acclimation types (HL-1, HL-2, and LL) across four nitrogen levels (N0, N100, N200, and N300); b, c, and d represent the HL-1, HL-2, and LL leaves, respectively, at different leaf positions (FL, 2L, and 3L) across four nitrogen levels (N0, N100, N200, and N300).

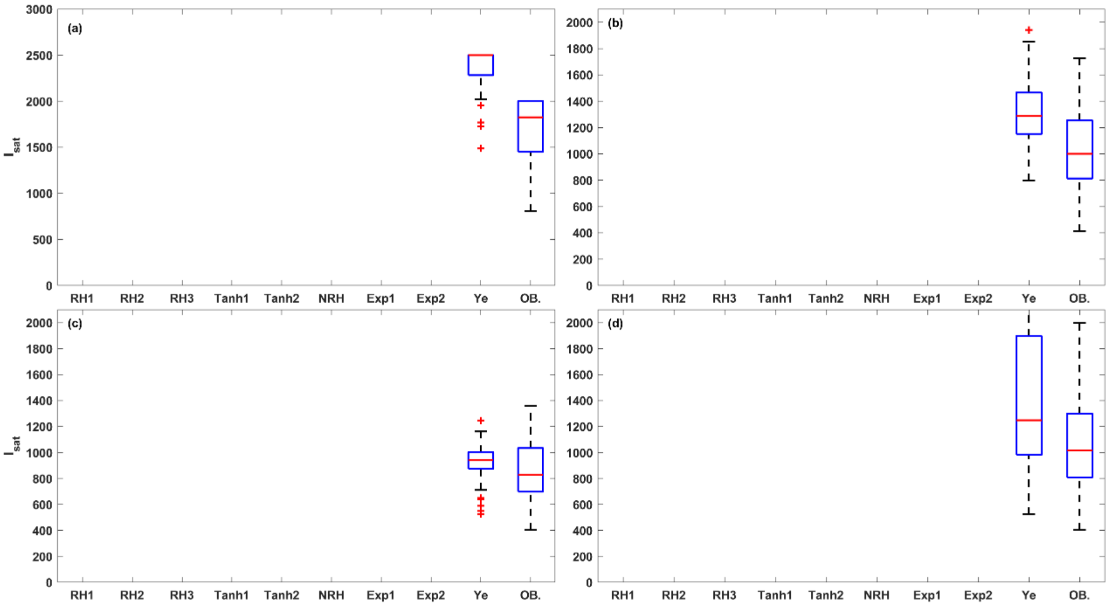

Figure S2: The boxplots of I_{sat} values estimated from the nine LRC models compared across HL-1 (a), HL-2 (b), and LL (c) and the overall set (d) (OB. denotes the observation of I_{sat}). Red + indicate outliers, defined as values beyond the minimum and maximum values within 1.5 times the IQR from the first and third quartiles.

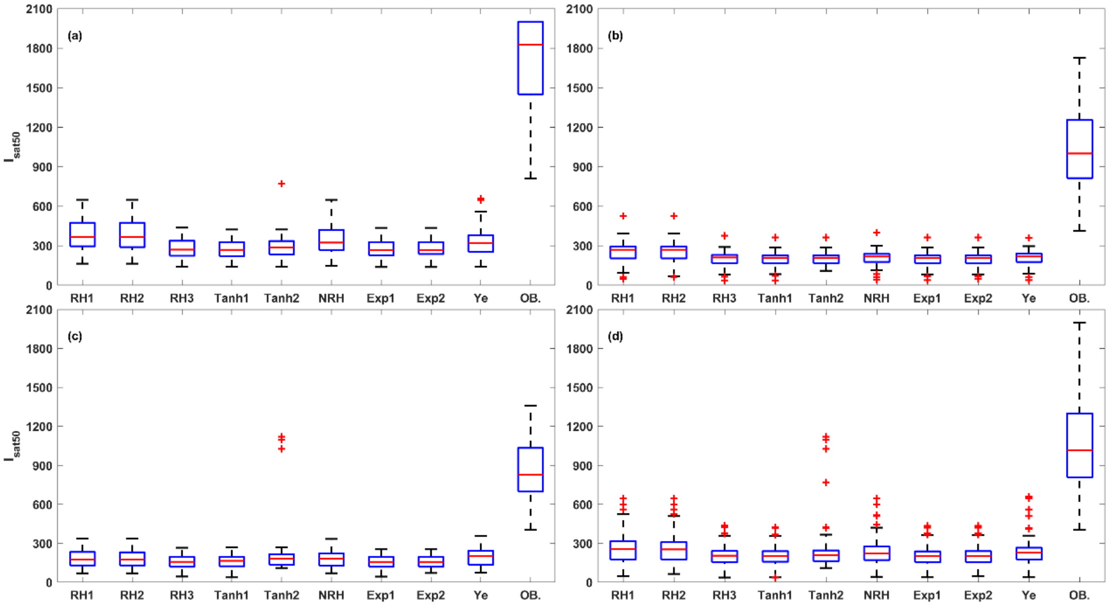

Figure S3: The boxplots of I_{sat50} values estimated from the nine LRC models compared across HL-1 (a), HL-2 (b), and LL (c) and the overall set (d) (OB. denotes the observation of I_{sat}). Red + indicate outliers, defined as values beyond the minimum and maximum values within 1.5 times the IQR from the first and third quartiles.

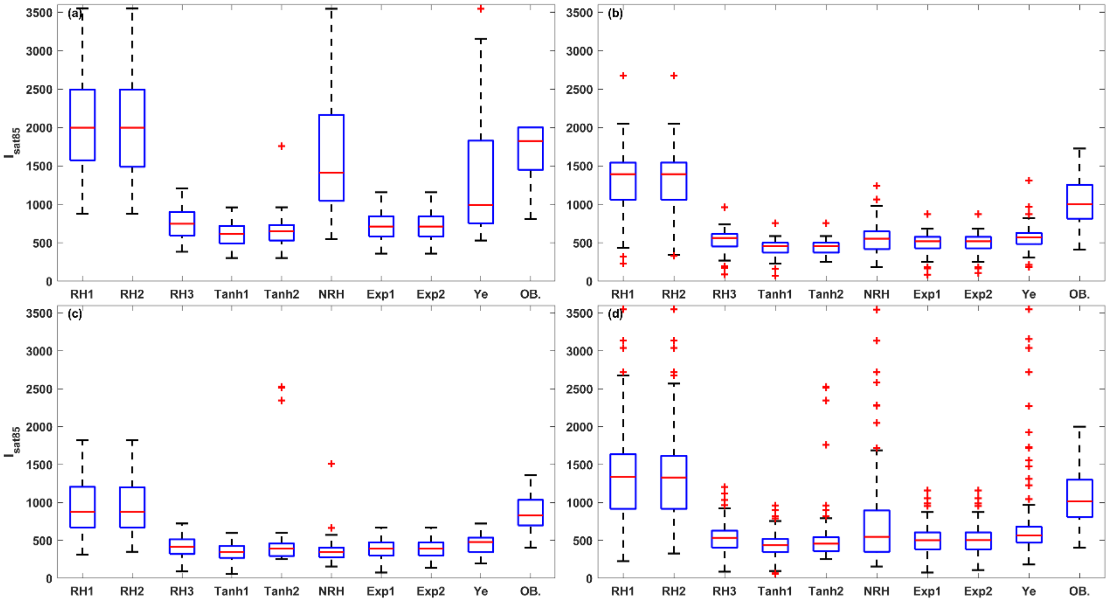

Figure S4: The boxplots of I_{sat85} values estimated from the nine LRC models compared across HL-1 (a), HL-2 (b), and LL (c) and the overall set (d) (OB. denotes the observation of I_{sat}). Red + indicate outliers, defined as values beyond the minimum and maximum values within 1.5 times the IQR from the first and third quartiles.

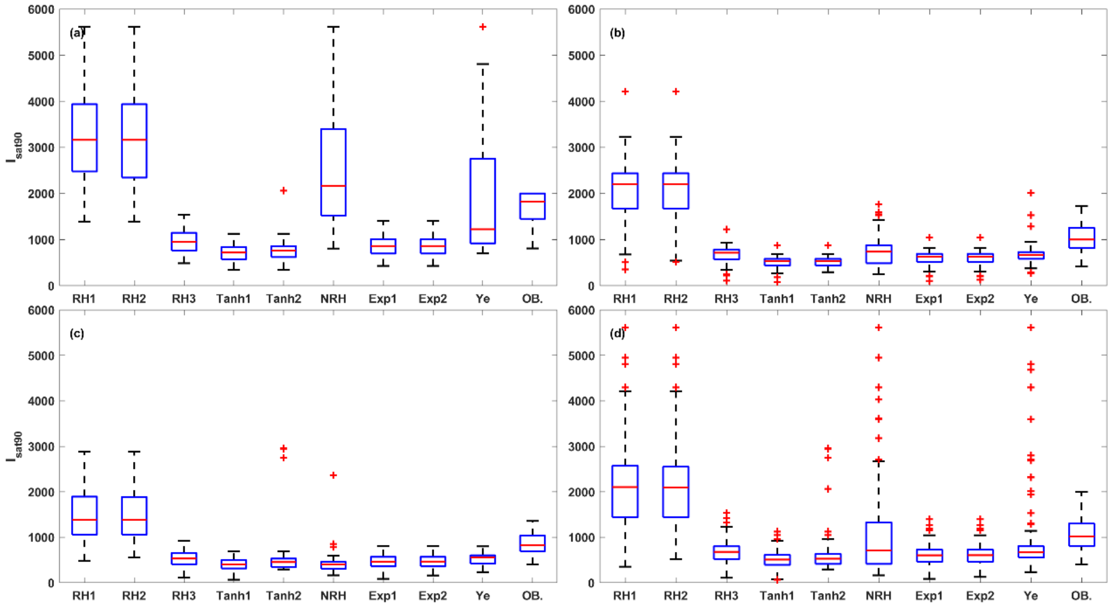

Figure S5: The boxplots of I_{sat90} values estimated from the nine LRC models compared across HL-1 (a), HL-2 (b), and LL (c) and the overall set (d) (OB. denotes the observation of I_{sat}). Red + indicate outliers, defined as values beyond the minimum and maximum values within 1.5 times the IQR from the first and third quartiles.

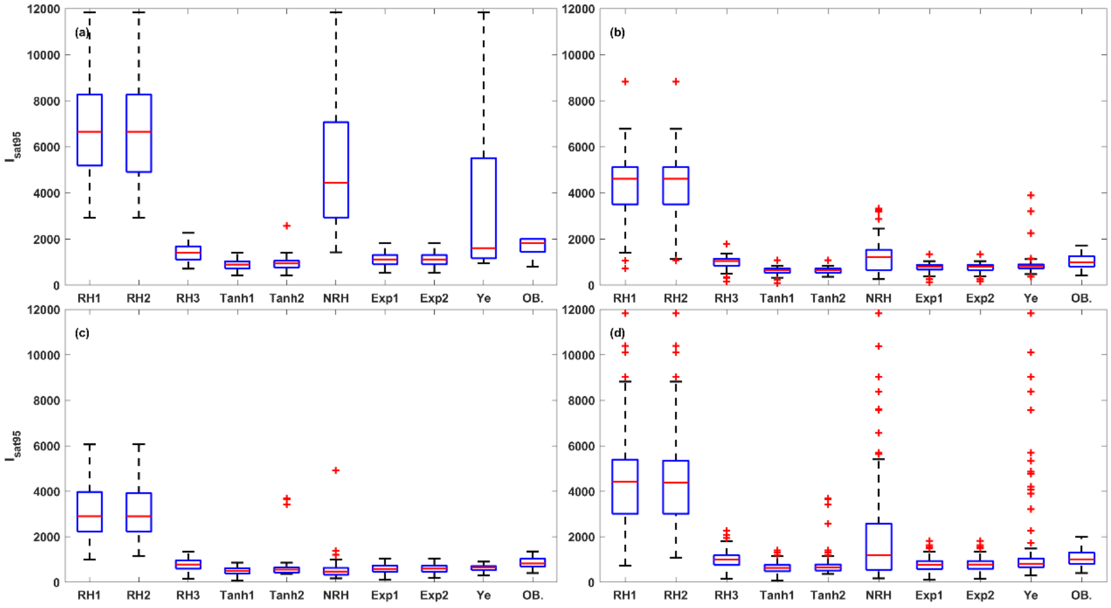

Figure S6: The boxplots of I_{sat95} values estimated from the nine LRC models compared across HL-1 (a), HL-2 (b), and LL (c) and the overall set (d) (OB. denotes the observation of I_{sat}). Red + indicate outliers, defined as values beyond the minimum and maximum values within 1.5 times the IQR from the first and third quartiles.

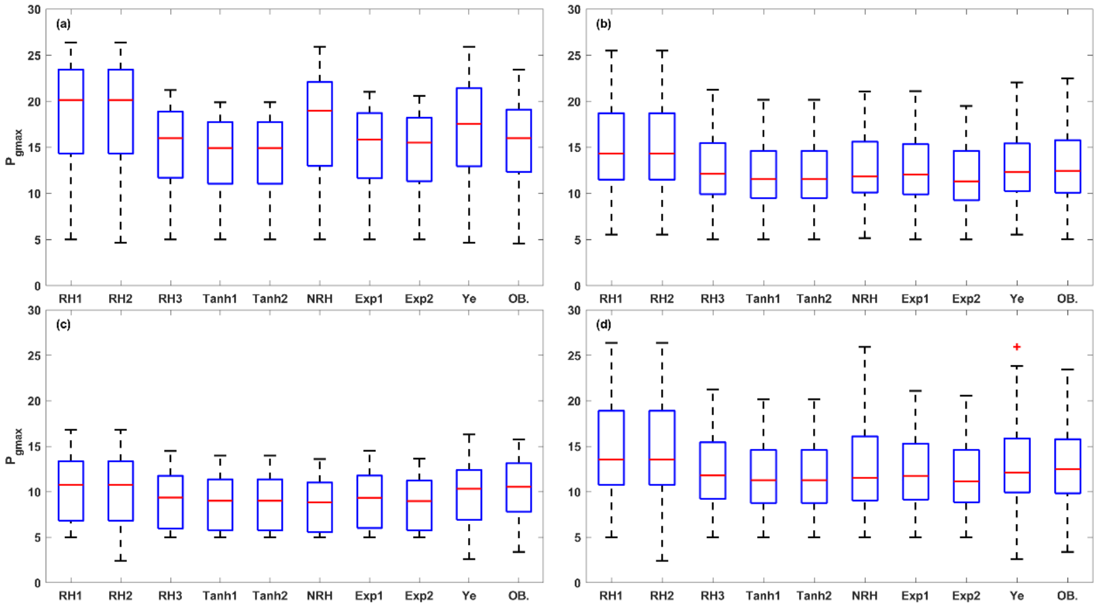

Figure S7: The boxplots of P_{gmax} values estimated from the nine LRC models compared across HL-1 (a), HL-2 (b), and LL (c) and the overall set (d), and compared with the observations of P_{gmax} (OB.). Red + indicate outliers, defined as values beyond the minimum and maximum values within 1.5 times the IQR from the first and third quartiles.

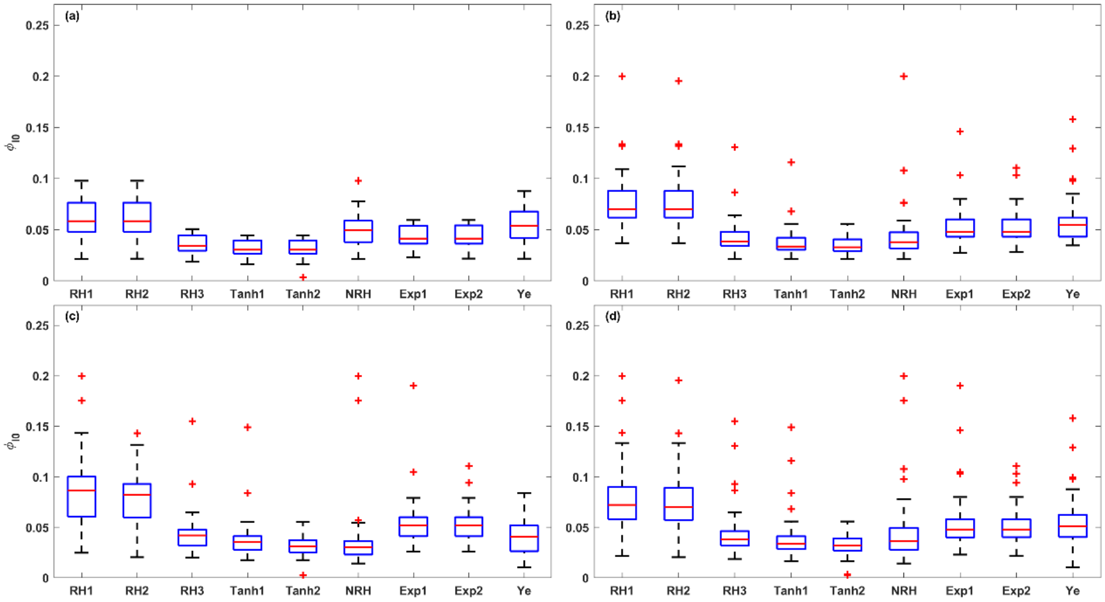

Figure S8: The boxplots of $\phi_{(I0)}$ values estimated from the nine LRC models compared across HL-1 (a), HL-2 (b), and LL (c) and the overall set (d). Red + indicate outliers, defined as values beyond the minimum and maximum values within 1.5 times the IQR from the first and third quartiles.

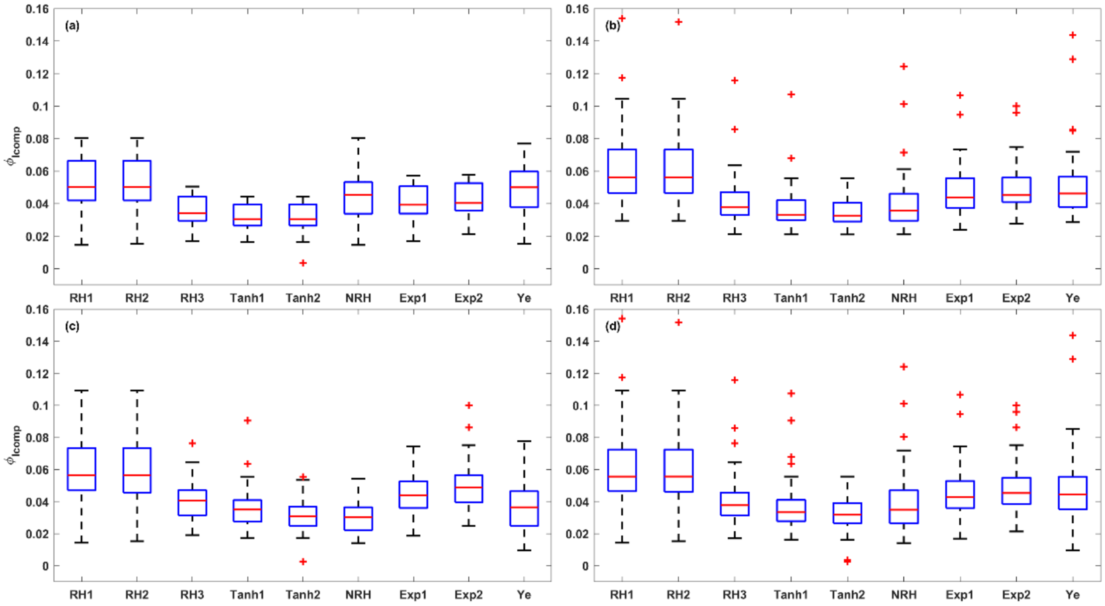

Figure S9: The boxplots of $\phi_{(Icomp)}$ values estimated from the nine LRC models compared across HL-1 (a), HL-2 (b), and LL (c) and the overall set (d). Red

+ indicate outliers, defined as values beyond the minimum and maximum values within 1.5 times the IQR from the first and third quartiles.

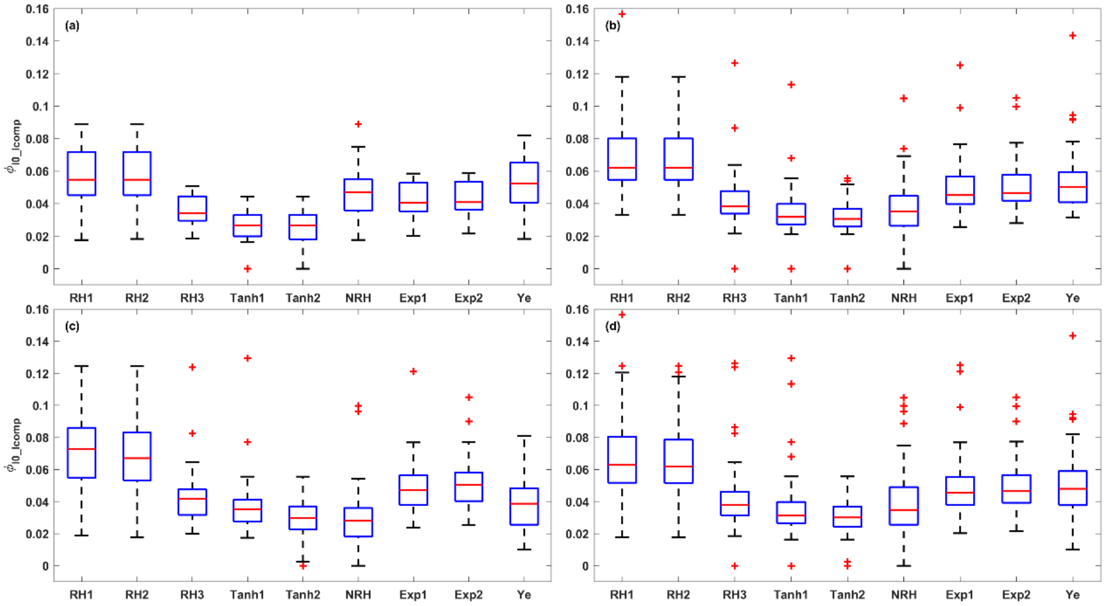

Figure S10: The boxplots of $\phi_{(I0\_Icomp)}$ values estimated from the nine LRC models compared across HL-1 (a), HL-2 (b), and LL (c) and the overall set (d). Red + indicate outliers, defined as values beyond the minimum and maximum values within 1.5 times the IQR from the first and third quartiles.

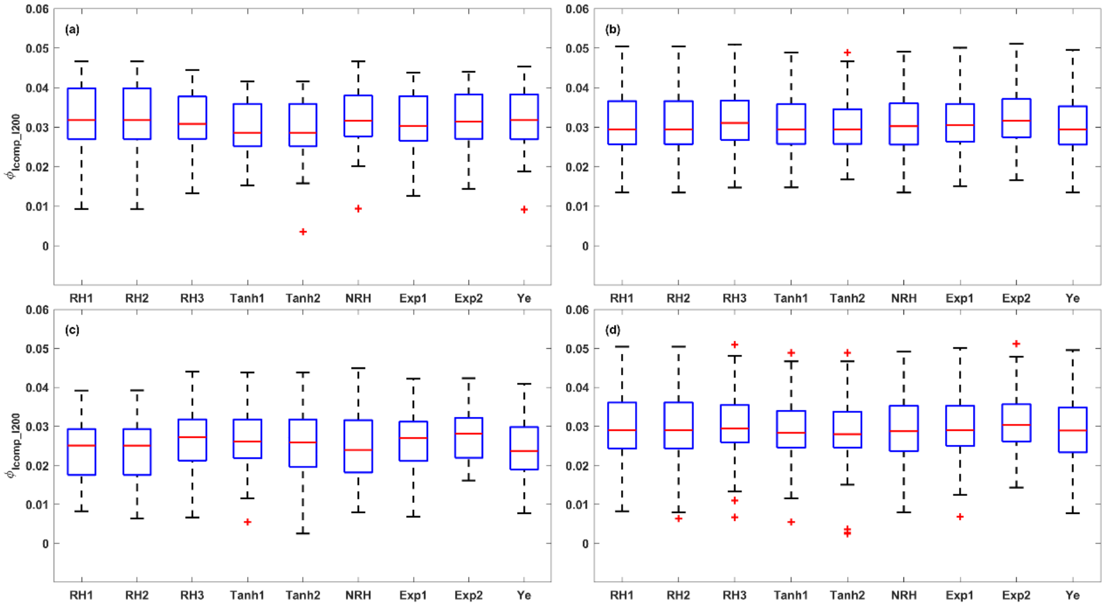

Figure S11: The boxplots of $\phi_{(Icomp\_I200)}$ values estimated from the nine LRC models compared across HL-1 (a), HL-2 (b), and LL (c) and the overall set (d). Red + indicate outliers, defined as values beyond the minimum and maximum values within 1.5 times the IQR from the first and third quartiles.
